# Advantages of Single-Molecule Real-Time Sequencing in High-GC Content Genomes

**DOI:** 10.1371/journal.pone.0068824

**Published:** 2013-07-23

**Authors:** Seung Chul Shin, Do Hwan Ahn, Su Jin Kim, Hyoungseok Lee, Tae-Jin Oh, Jong Eun Lee, Hyun Park

**Affiliations:** 1 Korea Polar Research Institute, Yeonsu-gu, Incheon, Korea; 2 University of Science & Technology, Yuseong-gu, Daejeon, Korea; 3 College of Life Sciences and Biotechnology, Korea University, Seongbuk-gu, Seoul, Korea; 4 Department of Pharmaceutical Engineering, SunMoon University, Asan, Korea; 5 DNALink, Inc. Songpa-gu, Seoul, Korea; Yale University, United States of America

## Abstract

Next-generation sequencing has become the most widely used sequencing technology in genomics research, but it has inherent drawbacks when dealing with high-GC content genomes. Recently, single-molecule real-time sequencing technology (SMRT) was introduced as a third-generation sequencing strategy to compensate for this drawback. Here, we report that the unbiased and longer read length of SMRT sequencing markedly improved genome assembly with high GC content via gap filling and repeat resolution.

## Introduction

Technical advances in DNA sequencing are key to the current capacity to complete organismal genomes, especially microbial genomes, rapidly and at low cost using next-generation sequencing (NGS) technologies such as Illumina Genome Analyser, SOLiD and Roche 454 platforms. However, the low cost and high-throughput of NGS are still insufficient for complete sequencing of genomes with high GC contents because this technology relies on a template amplification phase prior to sequencing, leading to biased coverage when using a high-GC genome as the template [1], [2], [3]. The Single-Molecule Real-Time (SMRT) sequencing technology recently developed by Pacific Biosciences (PacBio *RS*) avoids the amplification step and provides sequence data for individual template molecules, minimising the risk of introducing substitutions and/or low bias during amplification [4], [5]. Therefore, this method is expected to compensate for the major drawback of next-generation sequencing of high-GC content genomes.

PacBio *RS* is generally applied to two types of sequencing, i.e. Continuous Long Reads (CLRs) and Circular Consensus Sequencing (CCS) reads. CLR involves single-pass SMRT reads and ∼10 kb in length with only 82.1%–84.4% base accuracy [4]. CCS reads are consensus sequences obtained from multiple passes on a single sequence with relatively short read lengths (∼2 kb) and a low error rate [6]. Despite the low accuracy of CLRs, the longer read length and low bias have major advantages with regard to resolving complex repeats and filling the gaps in *de novo* assembly. Therefore, tools for correcting low-quality reads generated by PacBio *RS* have been developed, including LSC, p-errormodule of SMRT analysis (http://www.pacificbiosciences.com) and pacbioToCA [7], [8]. Using pacbioToCA, CLR sequences obtained by mapping high-quality short-read sequences were corrected with high-quality reads and achieved >99.9% accuracy. The statistics of assembly were markedly improved in *de novo* assembly with error-corrected reads. Also, CCS reads have been reported to improve yield and mean read length in comparison to Illumina short reads in error correction and in genome assembly with moderate GC content (http://www.pacificbiosciences.com).

Here, we evaluated the utility of the Pacific Biosciences *RS* platform for the sequencing of the high-GC content genome of *Streptomyces* sp., an endosymbiotic bacterium isolated from the Antarctic lichen *Cladonia borealis* with an estimated G+C content of 70.89%, to assess the advantages of unbiased single-molecule sequencing.

## Methods

### Genome sequencing

The endosymbiotic bacterium *Streptomyces* sp. PAMC 26508 was isolated from the Antarctic lichen *Cladonia borealis*. Genomic DNA for *Streptomyces* sp. PAMC 26508 was prepared according to Nikodinovic *et al*. [9]. For PacBio *RS* sequencing, two types of libraries were made with 1.5-kb and 8-kb sheared genomic DNA, and prepared using the standard PacBio *RS* sample preparation methods with C1 chemistry specific to each insert size. The 8-kb sample was sequenced on 1 SMRT cell with a 1×90 min collection protocol, and the 1.5-kb sample was sequenced on 8 SMRT cells with a 2×45 min collection protocol. A 300-bp paired end library for Illumina Hiseq 2000 and 7-kb paired end library for GS-FLX titanium were prepared, and sequencing was performed according to the manufacturers’ instructions. All sequencing processes were performed using the services of DNA Link, Inc.

### Error correction

The process of error correction was performed using the command pacBioToCA with the parameters -length 500 -partitions 200 -shortReads -l PAMC26508 -t 20 -s pacbio.spec [7]. CCS (26×length coverage) and Illumina (50×, 100× and 200× length coverage) reads were used for correction. Illumina reads were trimmed using FASTX-Toolkit (http://hannonlab.cshl.edu/fastx_toolkit) with the parameters -t 20 -l 50 -Q 33. Pacbio.spec file specified the parameter for overlapping the Illumina and pacbio data for correction: (i) utgErrorRate = 0.25; utgErrorLimit = 0.25; cnsErrorRate = 0.25; cgwErrorRate = 0.25; ovlErrorRate = 0.25; and merSize = 10. After correction, pacBio-corrected reads were analysed using FastQC (http://www.bioinformatics.babraham.ac.uk/projects/fastqc).

### Assembly and evaluation

Hybrid assemblies were performed using Celera Assembler modified to accept Continuous long reads of PacBio *RS* with the parameters (overlapper =  ovl unitigger =  bogart utgGraphErrorRate = 0.015 utgGraphErrorLimit = 2.5 utgMergeErrorRate = 0.030 utgMergeErrorLimit = 3.25 ovlErrorRate = 0.035 cnsErrorRate = 0.035 cgwErrorRate = 0.035 merSize = 28 doOverlapBasedTrimming = 1) [10]. Assembly evaluation was performed by using ALE, AMOS, HAWKEYE, MUMMER and BLAST [11], [12], [13], [14]. Different regions between assemblies were confirmed by PCR and Sanger sequencing. PCR primers were designed for the flanking region of integrase and tandem repeats in chromosome. Disagreements between the short-read assemblies and PBcR assemblies were further validated by PCR and Sanger sequencing. The used primers are shown Table S1.

### Contig ordering of the assembly PBcR_SR(50×)+CCS_+454, finishing of the genome, and circular map

To determine the order of contigs in the assembly PBcR_SR(50×)+CCS_+454, we designed primers for the flanking region of ribosomal DNA at the end of each contig and performed PCR using primer combinations. In addition, we used the sequences of the resulting PCR products to close all the gaps in the assembly PBcR_SR(50×)+CCS_+454. A circular map of contigs between assemblies and coverage plot of assembly with PBcR_SR(50×)+CCS_ was visualised using Circos [15]. Coverage value across the contigs was calculated using the command genomeCoverageBed of BEDTools [16].

### Data access

The raw data are available via NCBI. Accession numbers are SRA062237 for Short Read Archive, CP003990 for Chromosome, and CP003991 for Plasmid.

## Results

**Figure 1:**
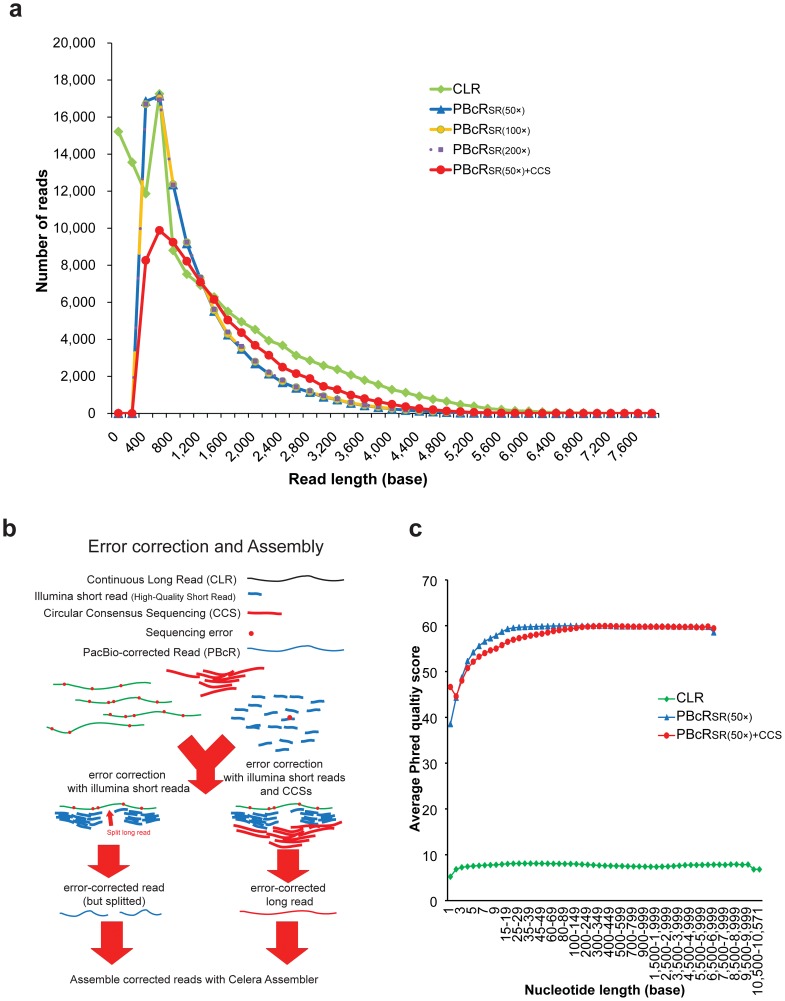
Statistics of error-corrected reads. (a) The length distribution of CLRs and PBcRs. Error correction of CLRs with Illumina short reads (50×, 100× and 200× coverage) showed similar length distributions. Larger numbers of Illumina short reads did not improve the results of error correction in the mean length of reads and throughput, but CCS reads increased both in mean length and throughput. (b) CCS increased the throughput of error correction by joining the break positions with no short-read coverage. (c) Base qualities of CLRs and PBcRs, where the x-axis correspnds to base position and the y-axis to the average Phred quality score.

**Figure 2:**
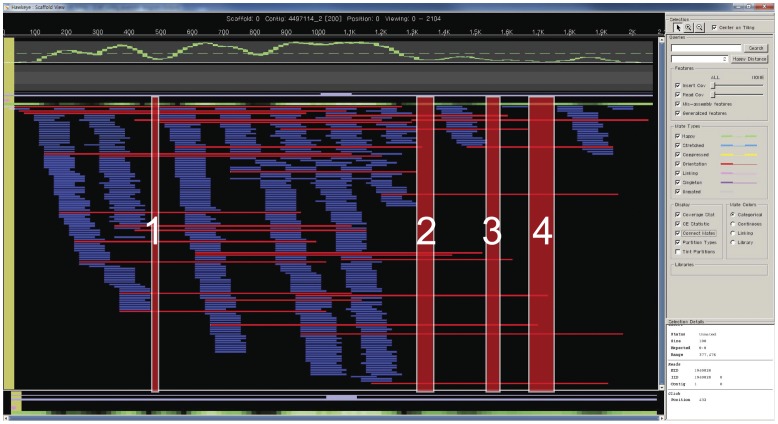
Results of error correction using 50× SR and 16× CCS reads. HAWKEYE indicated how to correct the errors of CLR with SRs (blue) and CCS reads (red). The numbers indicate the regions aligned with only CCS reads. CCS reads improved the throughput of error correction by spanning the unaligned region by SRs.

We combined three sequencing platforms: PacBio *RS*, GS-FLX titanium and Illumina Hiseq 2000 (Table 1). First, CLRs were corrected with high-accuracy sequences of Illumina or CCS reads with the pacBio-corrected Read (PBcR) algorithm [7]. Using 50× Illumina Short Reads (SRs) to correct 25× CLRs generated 14×corrected reads with the PBcR algorithm (Fig. 1a and Table 1). However, when we corrected CLRs with 100× and 200× SRs, additional SRs did not increase the mean read length or total bases. We examined whether unbiased CCS reads with improved sequencing accuracy could increase the throughput in error correction with high GC content and found that the addition of 26×CCS reads to 50×SRs in error correction increased throughput with 1×genome coverage and the average read length to 1.56 kb. In the PBcR algorithm, high-quality reads were aligned to CLRs, and the aligned regions were corrected with high quality. Then, CLRs were split into multiple fragments at unaligned regions. CCS reads improved the results through filling the regions that could not be aligned based on SRs (Fig. 1b, Fig. 2 and Table 2). After error correction, both CLRs corrected using 50×SRs (PBcR_SR(50×)_) and CLRs corrected using 50×SRs and 26×CCS reads (PBcR_SR(50×)+CCS_) showed high quality (>99.9%) (Fig. 1c). We also estimated the true accuracies of PBcR_SR(50×)_ and PBcR_SR(50×)+CCS_ by mapping to the contigs of the assembly SRs(100×)+454 with BLAST, and the estimate accuracy was 99.97% and 99.95%, respectively (Table S2).

**Table 1:** Sequencing statistics for *Streptomyces* sp. PAMC26508.

	Number of reads	Total bases	Mean read length (bp)	Coverage (X)
Illumina	18,000,000	1,687,126,990	94	222.0
454	291,450	58,354,948	200	7.7
CLR	132,907	187,805,069	1,413	24.7
PBcR_SR(50×)_	88,782	110,192,585	1,241	14.5
PBcR_SR(100×)_	89,183	111,502,750	1,250	14.7
PBcR_SR(200×)_	89,683	113,012,913	1,260	14.9
PBcR_SR(50×)+CCS_	78,388	122,272,261	1,560	16.1
CCS	253,467	197,046,247	777	25.9

**Table 2:** Split number of CLRs after error correction.

The number of split	PBcR_SR__(50×)_	PBcR_SR__(100×)_	PBcR_SR__(200×)_	PBcR_SR_ _(50×)+CCS_
1	57,798	58,617	59,337	68,936
2	11,454	11,282	11,226	4,219
3	2,192	2,178	2,161	318
4	326	314	306	15
5	38	40	35	0
6	1	2	2	0
Sum	71,809	72,433	73,067	73,488

**Figure 3:**
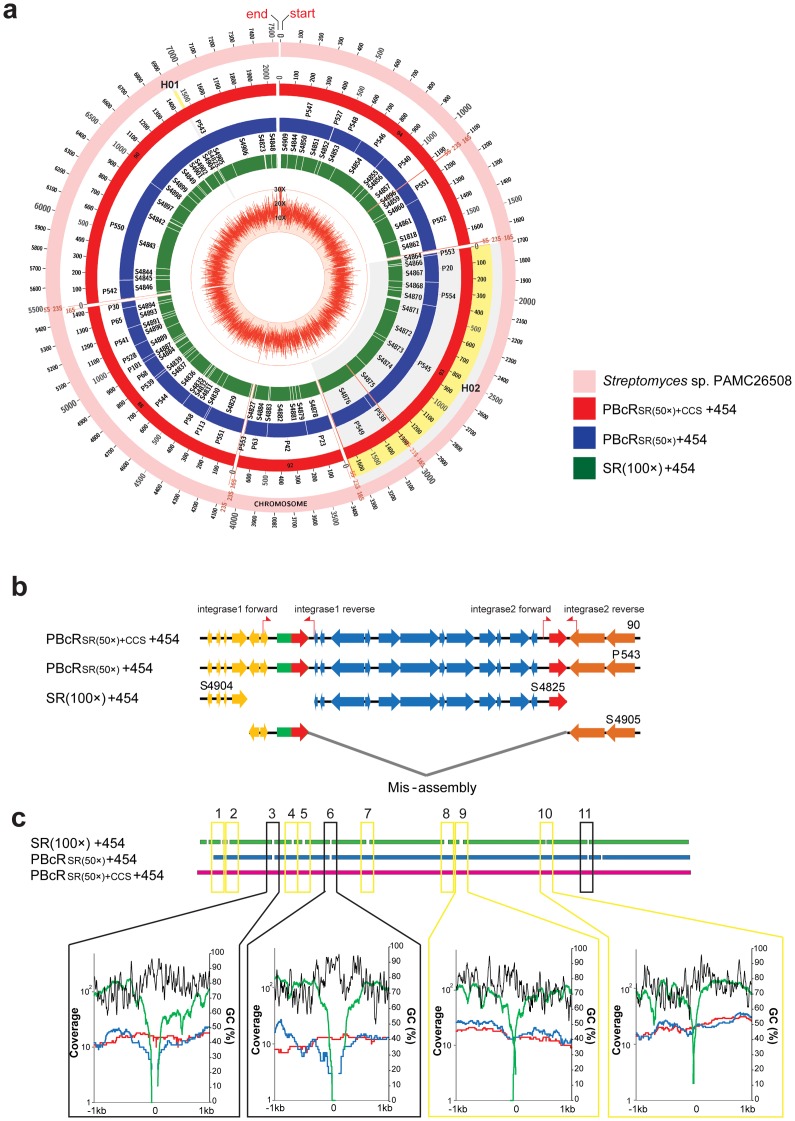
*Streptomyces* sp. PAMC 26508 assembly. (a) The outermost track (pink) represents the complete genome sequence of *Streptomyces* sp. PAMC 26508, the middle track (red) represents assembly with PBcR_SR(50×)+CCS_, the inner track (blue) represents assembly with PBcR_SR(50×)_ and the next track (green) represents assembly with SR. The innermost track (red line) indicates the read coverage of assembled contigs with PBcR_SR(50×)+CCS_. The numbers along the track indicate kilobase coordinates along the contig. The highlighted region H01 indicates the region of mis-assembled contig by repeat (Fig. 3b) and the highlighted region H02 indicates the representative region showing the differences in assemblies (Fig. 3c). (b) Red arrow indicates interspersed repeat sequences of the integrase gene. Contigs assembled from SRs(100×) with short read length were mis-assembled and split into three contigs by two integrase genes with identical sequences (600 bp long), but both PBcR_SR(50×)_ and PBcR_SR(50×)+CCS_ could resolve repeats due to their ability to span repeats. (c) The box indicates two types of gap: the black box indicates the gaps generated by assembly with both SRs(100×) and PBcRs reads, and the yellow box indicates the gaps generated by assembly with only SRs(100×) reads. Black line is GC content, and green, blue and red lines are each coverage, respectively. Each coverage and the average GC content for 25 base window of the flanking 1-kb region of gaps in assemblies. Gaps generated by assembly using short reads were filled with sufficient coverage of PBcRs, and PBcR_SR(50×)+CCS_ was able to span more gaps than PBcR_SR(50×)_. The local GC content of gaps is relatively higher than contigs.

We compared the results of assemblies using SRs and PBcR with Celera Assembler [10]; 8×454 reads, paired end library with an insert length of 7 kb, were used to produce longer and more accurate scaffolds in all assemblies (Table 3 and Fig. 3a). The SRs(200×)+454 assembly showed that use of more than 100×SRs could not increase the N50 contig size and filling the gap as much as the increment obtained in SRs, and also that the numbers of scaffolds and contigs were not increased. However, with assembly using only PBcR_SR(50×)_, the contig number was reduced by half (54 contigs) and the N50 contig size was increased to 410 kb compared with the assembly of SRs(100×)+454. In addition, using error-corrected PBcR with a combination of 50× SRs and 26× CCS reads, the assembled results using PBcR_SR(50×)+CCS_ reducing the contig nember to 6, 5 contigs comprising chromosome and 1 contig comprising a plasmid and increased the N50 contig size to 1.43 Mb compared with the assembly of SRs(100×)+454. Most ends of contigs comprising chromosome sequences corresponded to the region of ribosomal RNA operons (>5.7 kb long); so PBcR_SR(50×)+CCS_ (mean 1.56 kb) was shown to be too short for spanning this repeat region in this assembly.

**Table 3:** Assembly statistics for *Streptomyces* sp. PAMC26508.

Technology	Number of Contigs	Contig N50 (bp)	Average Contig Length (bp)	Max. Contig Length (bp)	Total Contig Length (bp)	Number of Scaff olds	Total Contigs In Scaff olds	Scaff olds N50 (bp)	Max. Scaff old Length (bp)
SRs(50×)+454	120	129,448	63,061	469,511	7,567,335	8	120	5,619,417	5,619,417
SRs(100×)+454	94	157,129	80,834	423,641	7,598,414	7	94	5,664,954	5,664,954
SRs(200×)+454	91	203,518	83,199	520,869	7,571,108	15	91	2,487,789	2,919,883
SRs(300×)+454	79	226,767	96,034	683,687	7,586,675	11	79	2,912,899	3,320,515
PBcR_SR(50×)_+454	54	410,617	142,250	1,112,582	7,681,514	27	54	2,398,168	2,995,774
PBcR_SR(50×)+CCS_+454 chromosome plasmid	6 1 1	1,430,884	1,272,366	2,055,222	7,634,199 7,526,197 104,048	5	6	3,486,126	3,486,126

**Figure 4:**
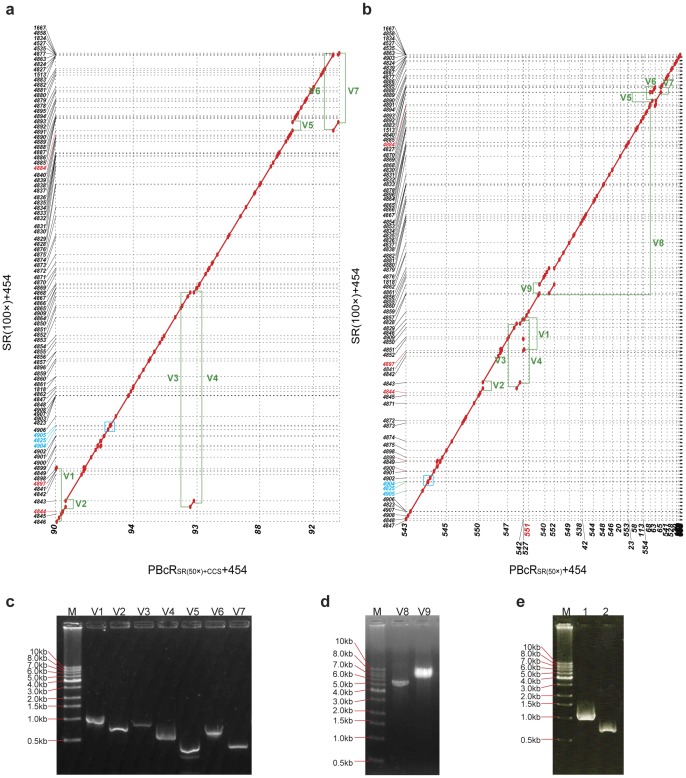
Dot plot showed that the assembly PBcR_SR(50×)+CCS_+454 was more accurate than other assembies. **SRs(100×)+454 to the contigs assembled with PBcRs.** (a) contigs of the assembly SRs(100×)+454 *vs*. contigs of PBcR_SR(50×)+CCS_+454. (b) contigs of the assembly SRs(100×)+454 *vs.* contigs of PBcR_SR(50×)_+454. Horizontal and vertical dotted lines indicate the boundaries of each contig. The red contig number indicate the mis-assembled contigs, and the blue contig number and rectangle indicate the region of mis-assembled contigs in Fig. 3b. (c) PCR validation of disagreements between Illumina short-read assembly and PBcR assembly (V1∼V7). Amplified V1∼V7 products showed that the contigs of the assembly SRs(100×)+454 were mis-assembled. (d) Contig 551 in the assembly PBcR_SR(50×)_+454 was confirmed to be mis-assembled in the region of ribosomal RNA operons with amplified V8 and V9 product. (e) The region of mis-assembled contig in Fig. 3b (indicated in blue rectangle of a and b) were validated by PCR: integrase 1 (lane1) and integrase 2 (lane2).

**Figure 5:**
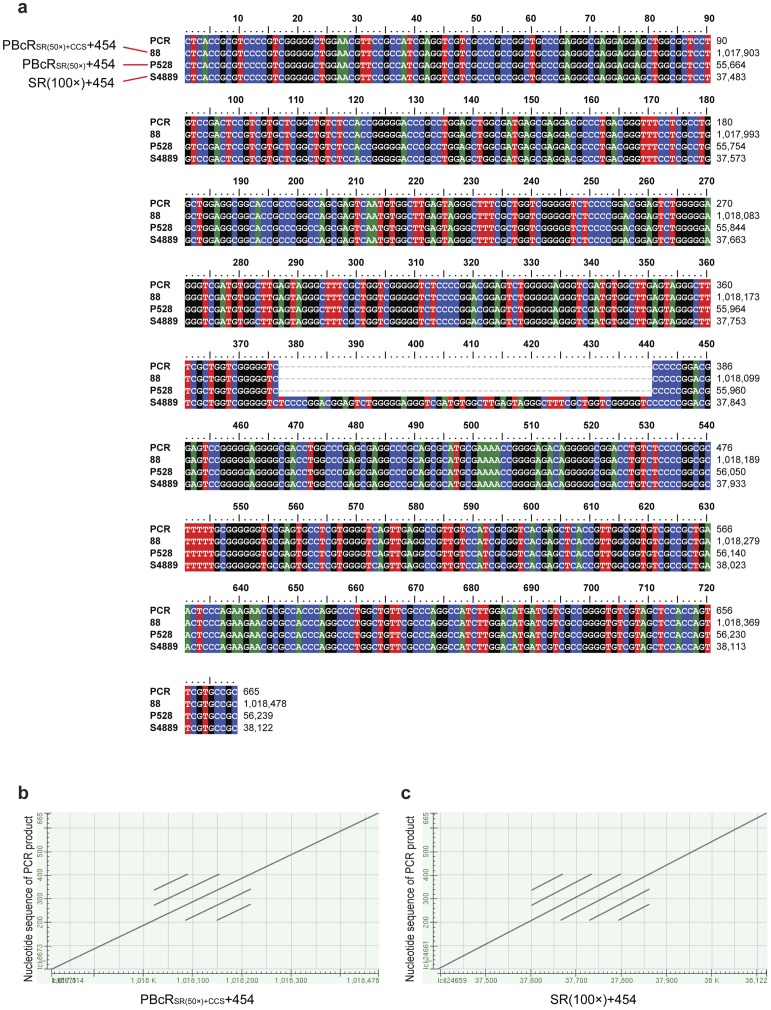
PBcRs resolved the collapsed tandem repeat in the chromosome of *Streptomyces* sp. PAMC26508. (a) The region of tandem repeats was amplified by PCR and sequenced. The tandem repeat was mis-assembled in the assembly SRs(100×)+454 due to the short length, but PBcRs resolved the tandem repeat by spanning the entire region. (b) The dot plot shows alignment of PCR product to the contig of PBcR_SR(50×)+CCS_+454. (c) The dot plot shows the alignment of PCR product to the contig of SRs(100×)+454.

We validated assemblies by aligning the contigs assembled using SRs to those assembled with PBcR_SR(50×)_ and PBcR_SR(50×)+CCS_ with the MUMmer sequence alignment tool (Fig. 4a and Fig. 4b). Although some regions of contigs showed different orders, the overall contig sequence identity of assemblies was estimated to be 99.99%. We also validated the regions showing disagreement in the alignment by PCR and Sanger sequencing (Fig. 4c, Fig. 4d, Fig. 4e and Figure S1), and demonstrate that PBcR with longer read length was more efficient for resolving interspersed and tandem repeats (Fig. 3b, Fig. 4e and Fig. 5). Assembly Likelihood Evaluation (ALE) framework, one of the recently pulished assembly likehood tools evaluating the accuracy of an assembly in a reference-independent manner, also showed that both PBcR and CCS increase the accuracy of an assembly (Figure S2).

Finally, we further investigated whether PBcR has an important role in gap filling in the assembly of a genome with high GC content. For example, in one of subset of the contigs, contig 93 of the assembly PBcR_SR(50×)+CCS_+454 was split into 4 contigs in the assembly PBcR_SR(50×)_+454 and 12 contigs in assembly SRs(100×)+454 (Fig. 3c). The graph of alignment coverage in the assembly SRs(100×)+454 showed that high-coverage Illumina reads could not fill the gaps between contigs, it appears that the high-GC content within the gaps led to bias in illumine sequence [1], but PBcR could fill these gaps with sufficient coverage (gaps 3, 6 and 11). These results indicated that unbiased SMRT sequencing may be sufficient to fill the gaps generated by next-generation sequencing technology in the assembly of a genome with high GC content.

## Discussion

Recently, most genome sequencing projects are carried out using automated applications of the NGS sequencing techniques, these newly developed methodologies may enable even more rapid bacterial genome sequencing. A genome project progresses through phases of data acquisition, assembly of the sequence reads, and then annotation and exploitation of the assembled data. During the data analysis, the de novo genome assemblies are potentially two incompleteness. First, Individual reads from NGS platform can have errors because they require amplification of source DNA before sequencing, leading to amplification artifacts and biased coverage of the genome, also, they have shown frequently incorrect read in homopolymer and/or very shot repeat regions. Second, they produce relatively short reads (median lengths of 100 bp for Illumina and about 700 bp for 454), it make assembly and related analyses difficult leading to transcript variants, although more computational power and several assembler has been developed. Recently, the Pacific Biosciences technology, which is based on single-molecule real-time (SMRT) DNA sequencing and the lack of amplification in the library construction step, provides a fundamentally new data type that provides the potential to overcome these limitations by providing significantly longer reads (now averaging >1 kb). Especially, Next-generation sequencing produces more gaps in the assembly of a genome with high GC content than in a genome with moderate GC content. Many could not readily be amplified by PCR, even if the regions of gaps were amplified, and could not easily be sequenced. We showed that PacBio *RS* SMRT sequencing is advantageous to resolve this problem in genome assembly with unbiased high-throughput sequencing and longer read length.

The genome of *Streptomyces* sp. PAMC 26508 has a 7,526,197 base pair linear chromosome with 70.89% GC content, and it contained 1 plasmid with 104,048 base pair. PacBio read data (PBcR and CCS) can fill the 88 gaps of high-GC repeat region with sufficient coverage, and also it has shown efficiently resolve interspersed and short tandem repeats, which it cannot overcome with high coverage NGS data.

In summary, the results of assembly with PBcR showed higher advantage to those of a genome with high GC content and repetition geneome structure. Moreover, CCS reads were important in improving assembly using CLRs in the error-correction process and assembly.

## Supporting Information

Figure S1 **Dot plots between sequence of PCR product and contig of the assembly PBcR_SR(50×)+CCS_ + 454 of** **Fig. 4c** **.**
(PDF)

Figure S2 **Validation of assemblies with assembly likehood tools evaluating the accuracy of an assembly in a reference-independent manner.**
(PDF)

Table S1 **Primer sequences.**
(PDF)

Table S2 **The identity of PBcR_SR(50×)+CCS_ in the assembly SR(100×)+454.**
(PDF)
