# Supplementary material for: Advantages of Single-Molecule Real-Time Sequencing in High-GC Content Genomes
Source: PLoS One. 2013 Jul 23;8(7):e68824. doi: 10.1371/journal.pone.0068824 (PMC3720884; doi:10.1371/journal.pone.0068824)
Supplement: Figure S1 — Dot plots between sequence of PCR product and contig of the assembly PBcRSR(50×)+CCS + 454 of Fig. 4c . (PDF) [file pone.0068824.s001.pdf]

Figure S1. Dot plots between sequence of PCR product and contig of the assembly PBcR$_{SR(50\times)+CCS}$ + 454 of Fig. 4c.

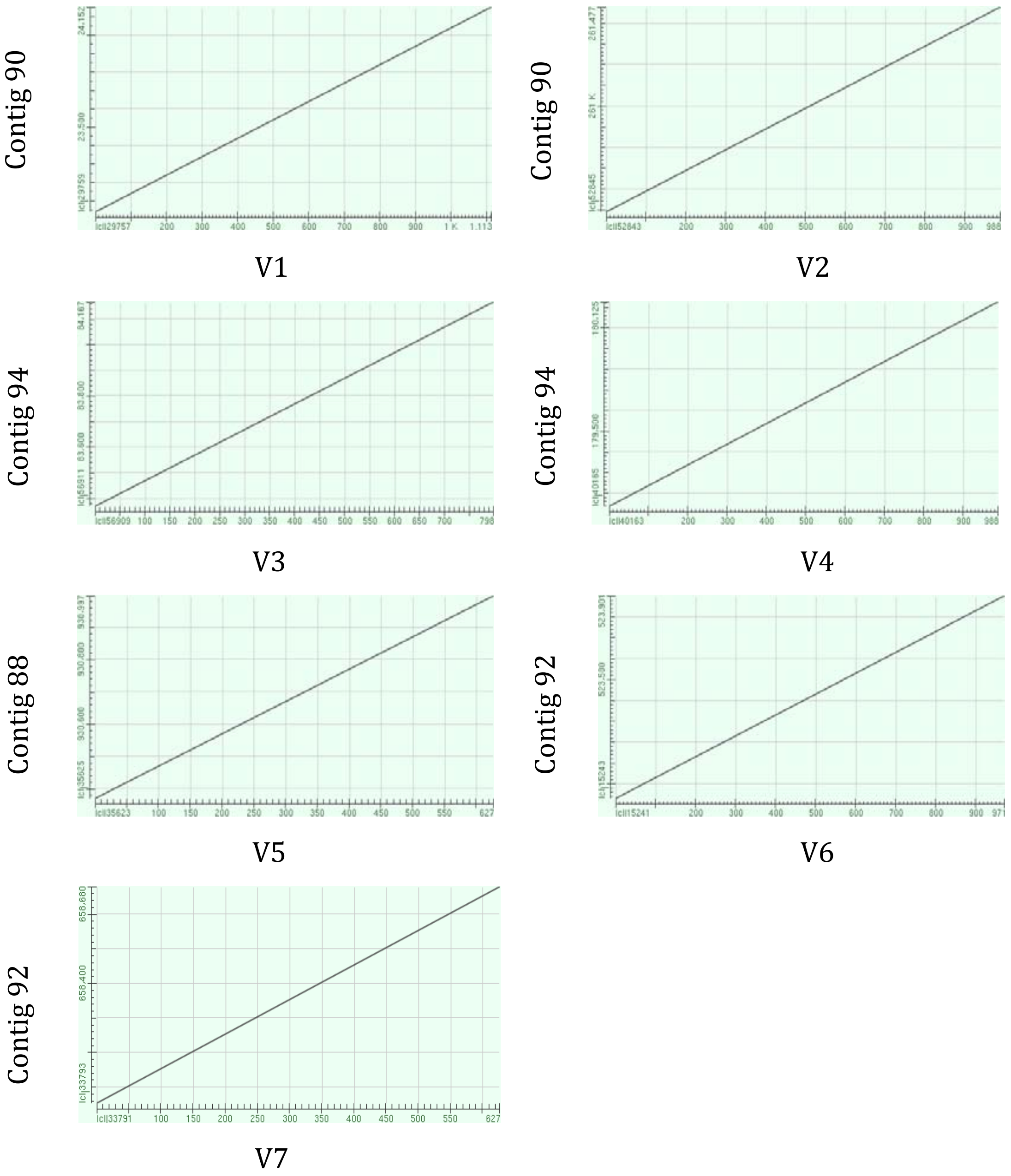

Dot plots show that mis-assemblies were occurred in the assembly SR(100×)+454, not in the assembly PBcR$_{SR(50\times)+CCS}$ + 454 (V1~V7). x-axis is the sequence of PCR product, and y-axis is the contig of the assembly PBcR$_{SR(50\times)+CCS}$ + 454.
