# Supplementary material for: Advantages of Single-Molecule Real-Time Sequencing in High-GC Content Genomes
Source: PLoS One. 2013 Jul 23;8(7):e68824. doi: 10.1371/journal.pone.0068824 (PMC3720884; doi:10.1371/journal.pone.0068824)
Supplement: Figure S2 — Validation of assemblies with assembly likehood tools evaluating the accuracy of an assembly in a reference-independent manner. (PDF) [file pone.0068824.s002.pdf]

Figure S2. Validation of assemblies with assembly likehood tools evaluating the accuracy of an assembly in a reference-independent manner.

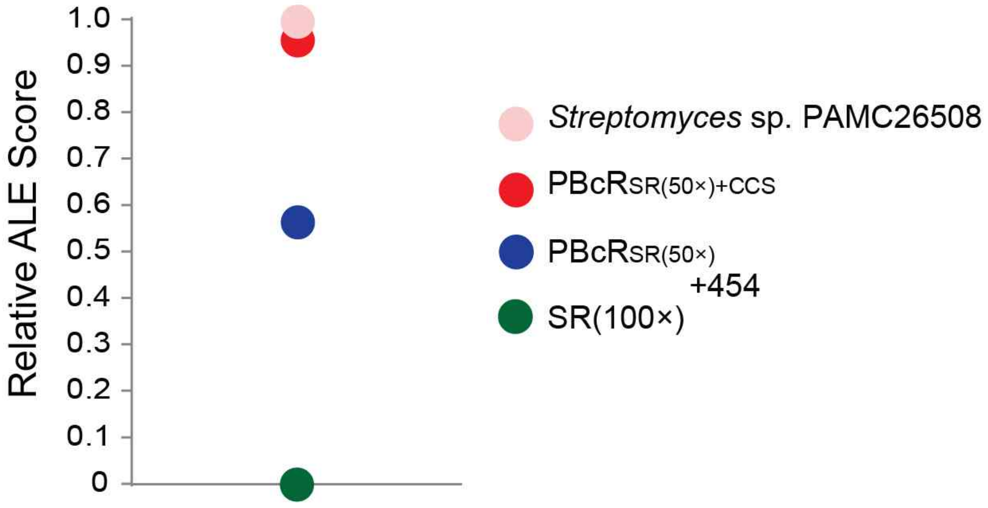

ALE scores indicate overall assembly accuracy. Because ALE scores decreases as the number of errors in assembly, the assembly PBcR$_{SR(50\times)+CCS}$ + 454 was evaluated as the best assembly with using ALE. ALE scores from each assembly were scaled to [0, 1] to get relative ALE scores.
