# Supplementary material for: Advantages of Single-Molecule Real-Time Sequencing in High-GC Content Genomes
Source: PLoS One. 2013 Jul 23;8(7):e68824. doi: 10.1371/journal.pone.0068824 (PMC3720884; doi:10.1371/journal.pone.0068824)
Supplement: Table S1 — Primer sequences. (PDF) [file pone.0068824.s003.pdf]

Table S1. Primer sequences

Primer names	Sequence (5´- 3´)
integrase1 forward	GCA CCC CGA ATG ATC AAA ATT CCG AAT G
integrase1 reverse	CGT GGT CTG CCG AGT GGT CTG GGC ACC C
integrase2 forward	GGA GTT GTT GTC GCC CTC GGG GCG CAG G
integrase2 reverse	CGG CGG TTA CAA GAA CTC CGG CAT CG
tandem1 forward	CTC ACC GCG TCC CCG TCG GGG GCT GGA A
tandem1 reverse	CGG CAC CGC GTA CGC GCA CTG CGG CAC G
V1 forward	GTC GCG GCG GTC GTG GTG ACG CAC C
V1 reverse	CCC GGG CAA GGA CGG CTG GCT CTC CG
V2 forward	GAC GTC CAC CAG CGT CCC CTC CTC C
V2 reverse	TTG TCG TCG GGG TCG GCG AGG ACG A
V3 forward	GCC GAG GTC AGG AAG ATC CCG GCC G
V3 reverse	GCC GAG GTC AGG AAG ATC CCG GCC G
V4 forward	TGA TCA CAT GAA CCC CTC GTT GCC T
V4 reverse	ACC GCC CAC CGC TTG GGC TGG ACT T
V5 forward	CCA CAT GAT CGA CAC CGG GAC CCC C
V5 reverse	CCG ACG TCG GCC CTC GAC CCG GAG C
V6 forward	CCG GAC CCC TGC ACG GTG CCT CCC TG
V6 reverse	CAG GGG CAG CAG CAG CCC GGA GGG CGA C
V7 forward	GCG TGG CGC GGC CGG GGA GGC CGG G
V7 reverse	CTG GCC CTC CAG GAC GTC CTT GAT C
V8 forward	GTT GGG GAT GGT GAG TAC TCG GTC CGT CTG AAC TC
V8 reverse	GCG GTG AGC CCG ATG GCG CGG CCG GCA AGA G
V9 forward	GCG GGC TCG ACA GTC ACA AGG ACC TGT A
V9 reverse	GTG GAG CGT GCG ATA CTG CCC GCC ATG T
