# Supplementary material for: Advantages of Single-Molecule Real-Time Sequencing in High-GC Content Genomes
Source: PLoS One. 2013 Jul 23;8(7):e68824. doi: 10.1371/journal.pone.0068824 (PMC3720884; doi:10.1371/journal.pone.0068824)
Supplement: Table S2 — The identity of PBcRSR(50×)+CCS in the assembly SR(100×)+454. (PDF) [file pone.0068824.s004.pdf]

Table S2. The identity of $PBcR_{SR(50×)+CCS}$ in the assembly SR(100×)+454

	Number of completely matched reads	Total bases (bp)	Mean identity (%)
$PBcR_{SR(50×)}$	54,224	60,690,635	99.974
$PBcR_{SR(50×)+CCS}$	39,767	54,927,553	99.954

To calculate the identity of $PBcR_{SR(50×)+CCS,}$ $PBcR_{SR(50×)+CCS}$ reads were mapped to the contigs of the assembly SR(100×)+454 with BLAST, and the reads falling completely within the contig were collected. Then we evaluate their accuracy with mis-matched bases and gaps in the result of BLAST.
